# Current landscape and future directions of neoantigen vaccines: A new era of personalized cancer immunotherapy

**DOI:** 10.1016/j.xinn.2026.101357

**Published:** 2026-03-28

**Authors:** Xinyue Wang, Shu Su, Siyi Tan, Xiaosu Chen, Lifeng Wang, Shuan Wang, Fangjun Chen

**Affiliations:** 1 Department of Oncology, Nanjing Drum Tower Hospital, Affiliated Hospital of Medical School, Nanjing University, 321 Zhongshan Road, Nanjing 210008, China; 2 The State Key Laboratory of Pharmaceutical Biotechnology, Chemistry and Biomedicine Innovation Center (ChemBIC), Division of Immunology, Medical School, Nanjing University, Nanjing 210093, China; 3 Department of Clinical Nutrition, Nanjing Drum Tower Hospital, Affiliated Hospital of Medical School, Nanjing University, 321 Zhongshan Road, Nanjing 210008, China

**Keywords:** neoantigen vaccines, personalized cancer immunotherapy, delivery systems, clinical trials, tumor microenvironment, neoantigen identification

## Abstract

At the forefront of cancer immunotherapy, neoantigens are tumor specific and highly immunogenic, and they have emerged as the key target for personalized oncology. Neoantigen vaccines have demonstrated promising immunologic responses and survival benefit in clinical trials with the combination of chemoradiotherapy and immune checkpoint therapy. This review provides a comprehensive synthesis of biological mechanisms and the next-generation screening approach of cancer neoantigens. We evaluate the current vaccine platforms and the design and vector of cancer neoantigen vaccines, and we additionally analyze strategies to overcome immunosuppressive tumor microenvironment challenges. We conducted a detailed analysis of ongoing and preliminary neoantigen vaccine clinical trials, summarizing existing challenges and areas requiring further improvement. Through an in-depth analysis of the latest research advances, we propose current optimization strategies for neoantigen vaccines, including novel screening models and innovative delivery approaches. Future directions for personalized cancer immunotherapy are outlined, with neoantigen vaccines positioned as pivotal enabling platforms.

## Public summary

• Neoantigens are tumor-specific and highly immunogenic targets for personalized cancer immunotherapy.
• Artificial intelligence (AI) improves neoantigen prediction, yet challenges remain in accuracy.
• Diverse vaccine platforms show promise in clinical trials.
• Combination therapies are crucial for enhancing antitumor efficacy of neoantigen vaccines.

## Graphical abstract

**Figure:**
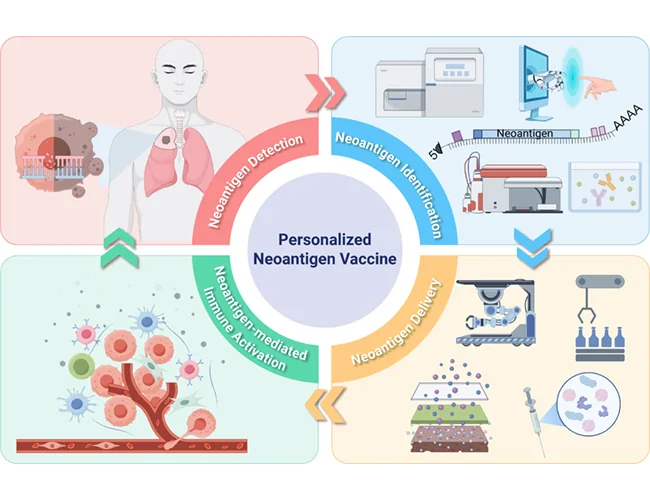

## Introduction

Neoantigens are tumor-specific peptides derived from somatic mutations that escape central tolerance and elicit potent antitumor immune responses.^1^ Unlike traditional tumor-associated antigens, neoantigens are exclusively expressed in tumor tissue, avoiding off-target effects and thus offering improved safety.^2^ It has been reported that the presence and functionality of neoantigen-specific T cells quantitatively predict the success of tumor immunotherapy, underscoring neoantigens’ critical role in antitumor immunity.^3^^,^^4^ With the advancement of new technologies, including high-throughput sequencing, AI, and bioinformatics, the approaches for neoantigen screening and identification have been steadily refined.^5^

Due to the escape from central immune tolerance, neoantigens demonstrate strong immunogenicity and potently activate immune responses, making them indispensable for personalized tumor vaccines.^6^ Neoantigen vaccines have made remarkable progress in clinical trials, effectively stimulating robust antitumor immune responses by targeting tumor-specific neoantigens. They show promise in shrinking tumors, prolonging survival, and improving patient outcomes, with personalized formulations tailored to individual mutations, representing a key advance in personalized cancer immunotherapy and highlighting their potential to transform cancer treatment. However, their clinical translation faces prominent bottlenecks.^7^ Firstly, neoantigen prediction accuracy is insufficient with high false-positive rates and lack of gold-standard validation.^8^ Secondly, tumor heterogeneity induces antigen escape via clonal evolution. Thirdly, delivery systems have low efficiency and limited immunogenicity, failing to trigger robust immune responses.^9^ Fourthly, clinical trials lack biomarker stratification, leading to significant efficacy variability. Based on the above bottlenecks, this review integrates interdisciplinary technological advances, analyzes the underlying causes of clinical trial success and failure, proposes precise translation strategies, and provides support for breaking through current dilemmas. Therefore, developing novel vaccine delivery systems and optimizing neoantigen vaccine design to enhance efficacy and reduce production costs represent key research priorities and unmet clinical needs.

This review comprehensively covers the biological mechanisms, screening methods, vector platforms, clinical progress in diverse cancers, and core bottlenecks of neoantigen vaccines. A key insight is that their advancement hinges on convergence science integrating computational biology immunology and materials science to break through technical barriers. It further proposes optimization strategies underscoring their pivotal role in personalized cancer immunotherapy.

## Basic principles of neoantigen presentation and immune recognition

### Immunological characteristics of neoantigens

Neoantigens are tumor-specific antigens derived from various somatic alterations, which occur exclusively in cancer cells. Their non-self nature endows them with high immunogenic potential.^10^ Genomic mutations represent a major source, including single-nucleotide variants (SNVs) that generate mutant peptides and insertions or deletions (indels) that frequently cause frameshift mutations producing novel open reading frames and highly immunogenic peptides.^11^ Gene fusions resulting from chromosomal rearrangements, such as BCR-ABL or EML4-ALK, yield unique and strongly immunogenic neoantigens with novel junctional epitopes completely absent in normal tissues. Beyond these, genomic structural variations including translocations, inversions, and large deletions create chimeric proteins that provide multiple potential major histocompatibility complex (MHC)-binding epitopes with high tumor specificity due to substantial deviations from the normal proteome.^12^

At the transcriptomic level, aberrant RNA splicing—including exon skipping, intron retention, and non-canonical exitron splicing—produces tumor-specific isoforms and neojunctions.^13^ Notably, emerging evidence reveals that long non-coding RNAs (lncRNAs), previously considered non-coding, can undergo translation to generate immunogenic peptides that are presented by MHC molecules, significantly expanding the neoantigen repertoire beyond protein-coding regions.^14^ RNA editing, particularly adenosine-to-inosine changes, introduces non-synonymous substitutions that create immunogenic peptides. Additionally, readthrough events caused by deletions of stop codons or 3′ untranslated regions (UTR) produce extended protein sequences with tumor-specific C-terminal alterations.^15^ Proteomic variants such as abnormal post-translational modifications and proteasome-catalyzed peptide splicing further contribute to the diversity of neoantigens. In virus-associated cancers, viral proteins serve as potent neoantigens due to their foreign origin.^16^

A key immunological characteristic of neoantigens is their absence in normal tissues, allowing them to evade central and peripheral tolerance mechanisms. Their immunogenicity is governed by efficient processing and presentation via MHC molecules, strong binding affinity to MHC, and successful engagement of T cell receptors without prior tolerance.^17^ Neoantigens derived from frameshifts, fusions, structural variations, and translated lncRNAs exhibit particularly high immunogenicity due to their significant divergence from self-proteins. These properties make neoantigens promising targets for personalized immunotherapies, including cancer vaccines, adoptive T cell transfer, and bispecific antibodies, as they elicit robust and specific T cell responses against tumors while minimizing off-target toxicity, although their variable prevalence across tumor types presents challenges for broad clinical application.^18^ Key differences in definition, tumor specificity, and limitations between these tumor antigen classes are summarized in Table 1.

**Table 1:** Definitions and key characteristics of major tumor antigen classes

Parameter	Shared tumor-associated antigens	Virus-encoded antigens	Neoantigens
Definition	self-derived antigens (overexpressed or differentiation-restricted) shared across patients, not tumor exclusive	antigens encoded by oncogenic viruses in virus-driven tumors, shared in virus-positive patient cohorts	patient-specific antigens derived from tumor somatic mutations, absent in normal tissues
Tumor specificity	moderate	high	high
Immunogenicity	weak	strong	strong
Tumor expression frequency	high	high	heterogeneous (variable across patients/tumors)
Current limitations	potential on-target off-tumor toxicity	limited to small subsets of virus-driven tumors	immature neoantigen screening and validation technologies

This review focuses primarily on neoantigen vaccines and broadens to shared tumor-associated antigens (TAAs) and virus-encoded antigens only in specific scenarios, including cross-antigen combination strategies to enhance immune response breadth and immunotherapy for virus-driven tumors, with explicit clarification of their relevance to neoantigen-based approaches.

### Core mechanisms of neoantigen-mediated immune activation

Neoantigen-mediated antitumor immunity constitutes a precisely orchestrated cascade, grounded in the unique tumor specificity of these antigens and their inherent capacity to bypass central tolerance. Originating from somatic mutations (e.g., SNVs, indels, gene fusions) exclusive to cancer cells, neoantigens escape negative selection in the thymus. This fundamental distinction from self-antigens underpins their potent immunogenicity.

The activation cascade is initiated by the release and presentation of neoantigens. This can occur through spontaneous or therapy-induced tumor cell death or via phagocytosis by professional antigen-presenting cells (APCs), most notably dendritic cells (DCs). Subsequent intracellular processing follows distinct pathways: proteasomal degradation generates short peptides (typically 8–11 amino acids) that are transported into the endoplasmic reticulum by the transporter associated with antigen processing (TAP) and loaded onto MHC-I molecules for presentation to CD8^+^ T cells. Alternatively, longer peptides may be cross-presented and loaded onto MHC-II molecules for recognition by CD4^+^ T cells.

A critical checkpoint in this process is T cell priming, which requires the integration of two signals. Signal 1 is antigen specific, delivered through T cell receptor (TCR) engagement with the neoantigen-MHC complex (MHC-I for CD8^+^ T cells, MHC-II for CD4^+^ T cells). Signal 2 is provided by co-stimulatory molecules (the CD28-B7 interaction), which is essential to prevent T cell anergy and outweigh concurrent co-inhibitory signals mediated by pathways such as programmed death-1 (PD-1)/programmed death-ligand 1 (PD-L1) and cytotoxic T-lymphocyte-associated protein 4 (CTLA-4).

Upon successful activation, cytotoxic CD8^+^ T lymphocytes (CTLs) migrate to the tumor microenvironment (TME) via chemokine gradients. They recognize neoantigen-MHC-I complexes on tumor cells and execute killing primarily through granule exocytosis (perforin/granzyme-mediated apoptosis) and Fas-FasL signaling. Concurrently, activated CD4^+^ T cells differentiate into helper subsets (e.g., Th1, Th17). They secrete cytokines such as interferon-γ (IFN-γ) and interleukin (IL)-2 to amplify CTL proliferation and enhance APC maturation. Additionally, these cytokines suppress immunosuppressive cells within the TME, including regulatory T cells (Tregs) and M2-type tumor-associated macrophages (TAMs).

A pivotal long-term outcome of this response is the establishment of immunological memory. Memory T cells, including central (Tcm) and effector (Tem) subsets, persist post clearance, enabling rapid recall responses upon tumor recurrence. However, this multi-step cascade is vulnerable to disruption at several key bottlenecks. These include impaired antigen processing or presentation (e.g., TAP dysfunction, MHC downregulation), T cell exhaustion, and the broadly immunosuppressive TME. These limitations directly inform therapeutic strategies—for instance, employing stimulator of interferon genes (STING) agonists to enhance APC function or PD-1 inhibitors to reverse T cell exhaustion.

In summary, this detailed mechanism bridges genomic instability in cancer to a targeted adaptive immune response, providing the essential theoretical foundation for the rational design of neoantigen vaccines and their combination with other immunotherapies.

### The impact of neoantigen on tumor evolution and prognosis

Neoantigen heterogeneity, manifesting as diversity both within and between tumors, is a pivotal characteristic with significant implications for cancer immunotherapy.^19^ Intratumorally, genomic instability drives the emergence of diverse mutations, such as SNVs, indels, gene fusions, and rearrangements. These mutations generate a wide array of neoantigens among tumor subclones. For example, in gliomas, high intratumor neoantigen heterogeneity leads to numerous subclonal neoantigens, impeding effective immune responses as T cells struggle to target a scattered set of antigens.^20^ Intertumoral heterogeneity is also pronounced.^21^ Different patients' tumors, even of the same histological type, can harbor distinct mutational landscapes, resulting in unique neoantigen profiles. Lung adenocarcinomas in various patients may have diverse driver mutations such as epidermal growth factor receptor (EGFR) and ALK, each associated with specific neoantigens.^22^ This heterogeneity poses challenges for developing universal immunotherapies, as standardized approaches may miss most neoantigens in a portion of patients.

Clonal neoantigens, which are shared across all tumor cells, represent prime targets for immunotherapy because they enable widespread immune-mediated destruction. These neoantigens arise from early driver mutations linked to stemness and induce robust systemic immune responses associated with durable clinical benefits.^23^ For example, clonal neoantigens derived from TP53 or KRAS mutations in pancreatic cancer have been linked to improved efficacy of checkpoint inhibitors, as T cells targeting these antigens can eliminate the entire tumor mass.^24^ In contrast, subclonal neoantigens, restricted to specific tumor subpopulations, present significant therapeutic challenges. Branching subclones, which remain unaffected by the antigen profiles of other clones, contribute to immune evasion. Targeting subclonal neoantigens alone often fails to eradicate the primary tumor, increasing the risk of relapse.^25^ However, combining subclonal antigens with clonal ones may enhance therapeutic efficacy. Tandem or combinatorial mutations, particularly in subclones exhibiting stem-like properties, can generate unique epitopes. Strategies that leverage both clonal and subclonal neoantigens—such as multi-epitope vaccines or engineered T cells targeting concatenated mutations—help address tumor heterogeneity.^26^ This dual approach targets dominant clones through clonal neoantigens while simultaneously eliminating resistant subclones, thereby reducing the likelihood of relapse and improving long-term therapeutic outcomes.

### Prediction and screening of neoantigens

Advances in neoantigen screening technologies, particularly single-cell sequencing,^27^ AI-based epitope prediction,^28^ and high-sensitivity mass spectrometry, are accelerating neoantigen vaccine development by enabling more accurate identification of immunogenic targets and addressing key challenges in tumor heterogeneity and antigen selection. High-throughput sequencing (HTS) is pivotal in neoantigen screening. Whole-exome sequencing compares tumor and normal exomes, pinpointing somatic mutations (e.g., SNVs, indels) encoding potential neoantigens.^29^ Transcriptome sequencing (RNA sequencing [RNA-seq]) identifies expressed mutations, ensuring candidates are transcribed and translated. Together, they prioritize mutations with transcriptional activity, filtering non-expressed variants. For example, in lung cancer, HTS identifies KRAS mutations via exome sequencing, while RNA-seq confirms their expression, validating them as neoantigen candidates. This integration streamlines neoantigen discovery by linking genomic mutations to functional expression.

**Figure 1:**
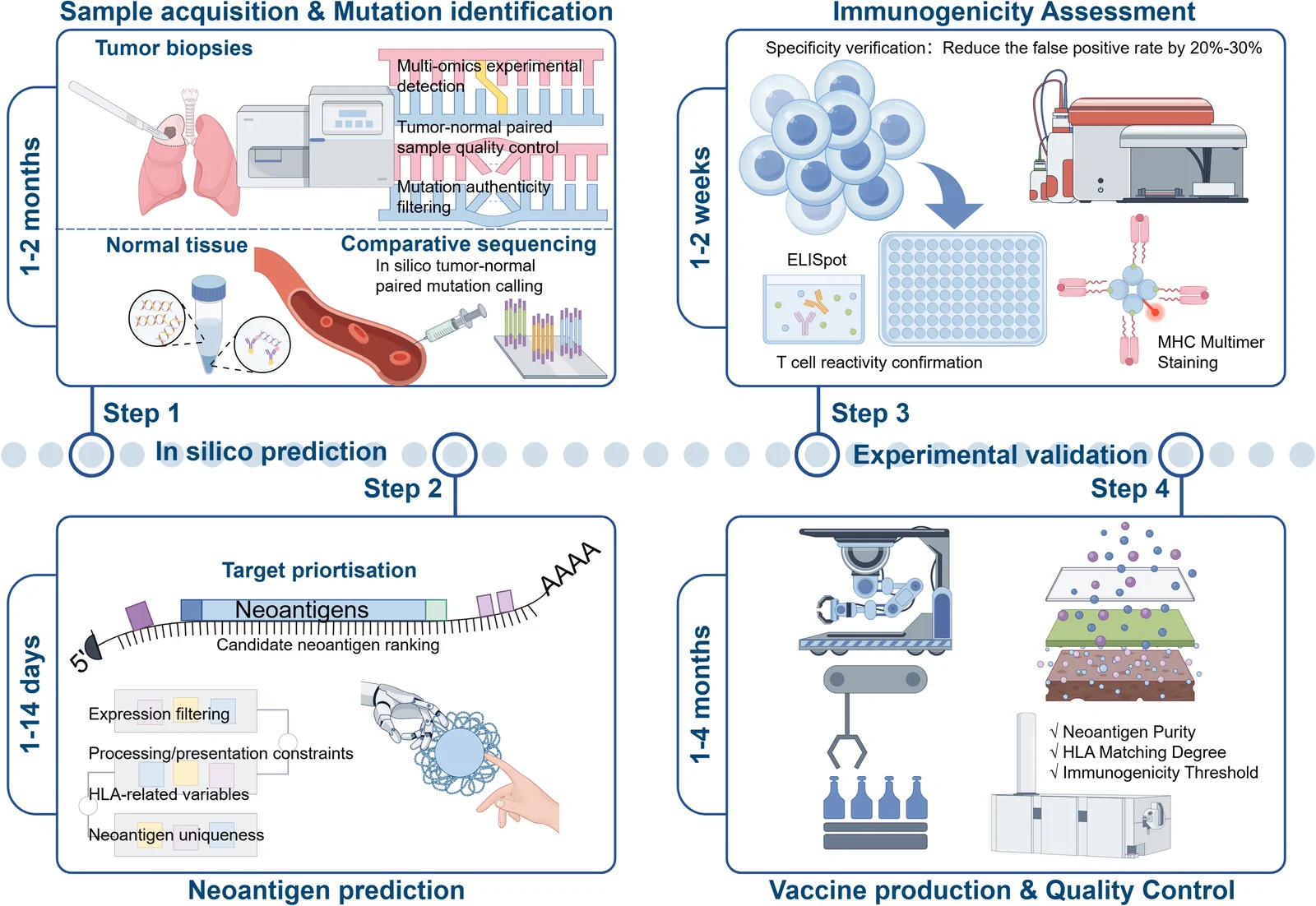
Manufacturing workflow for developing a personalized neoantigen cancer vaccine The process initiates with the collection of patient-derived tumor and matched normal biopsies (step 1). For these samples, multi-omics profiling (genomics, transcriptomics, immunopeptidomics) is performed alongside comparative sequencing to identify tumor-specific somatic mutations (the molecular basis of neoantigens), and this step typically takes 1–2 months. Subsequent to mutation identification, computational neoantigen prediction and target prioritization are conducted (step 2), where high-throughput epitope screening and preliminary immunogenicity evaluation can be substantially accelerated through AI approaches. This stage is completed within 1–14 days. Next, experimental immunogenicity assessment is carried out (step 3), including functional assays such as ELISpot and MHC multimer staining, which helps reduce the false-positive rate of predicted neoantigens by 20%–30%. This validation step requires 1–2 weeks. Finally, selected neoantigens are synthesized, incorporated into the vaccine formulation, and subjected to strict quality-control checkpoints (e.g., antigen purity, HLA matching degree, immunogenicity threshold) (step 4). Vaccine production and quality assurance take 1–4 months. The entire procedure from tissue acquisition to vaccine generation typically requires several months. Neoantigen, tumor-specific mutant epitopes recognizable by the host immune system; HLA, human leukocyte antigen (key antigen-presentation molecules and the target of HLA-matching degree in vaccine quality control); ELISpot, enzyme-linked immunospot assay (the step-3 technique for detecting cytokine secretion to confirm antigen-specific T cell reactivity); MHC, major histocompatibility complex.

Despite significant advances in genomic and bioinformatic technologies, the rapid and cost-effective identification of patient-specific immunogenic neoantigens remains a major obstacle in the development of personalized cancer immunotherapy (Figure 1). To address this challenge, Chen et al. established a novel strategy by constructing an inventory-shared neoantigen peptide library derived from recurrent hotspot mutations in common solid tumors—such as KRAS, TP53, and PIK3CA—and designed to target high-frequency human leukocyte antigen (HLA) class I alleles including HLA-A02, A11, and A∗24.^30^ This pre-screened, off-the-shelf library allowed for rapid matching with patient-specific mutation and HLA profiles obtained via clinical targeted sequencing, reducing the neoantigen identification timeline to approximately 10 days and substantially lowering synthesis and validation costs compared to *de novo* peptide design. However, this approach is limited by its lack of personalization, as it only covers patients whose tumors carry the included hotspot mutations and who express the common HLA alleles pre-selected in the library. This method can be extended to tumor-associated antigen (TAA) prediction, yet this review focuses on its application in neoantigen screening.

A sequential algorithmic workflow is essential for effective neoantigen screening. Mutation identification algorithms such as MuTect2 detect somatic variants from sequencing data, flagging potential neoantigen sources such as SNVs and indels by analyzing genomic differences between tumor and normal cells and filtering out germline variations to focus on tumor-specific changes.^31^ Next, MHC-binding prediction tools including NetMHCpan rank peptides by their affinity for specific HLA alleles, filtering high-affinity candidates that are likely to be presented on cell surfaces by assessing how well mutated peptides fit into MHC molecules.^32^^,^^33^ Finally, immunogenicity assessment algorithms such as DeepImmuno predict T cell recognition by integrating features such as peptide stability and TCR repertoire overlap, evaluating whether MHC-presented neoantigens can stimulate T cell activation beyond mere binding.^34^ Together, these algorithms prioritize actionable neoantigens by identifying relevant mutations, predicting their presentation, and scoring immunogenicity, streamlining preclinical validation.

Recent advances in next-generation multi-modal AI have significantly promoted neoantigen screening for cancer immunotherapy. This technology addresses two core pillars of T cell antigen identification: neoantigen-HLA binding and neoantigen-TCR interactions. Early computational methods were limited by allele/antigen specificity and inability to capture non-linear patterns—e.g., SYFPEITHI^35^ and MixMHCpred^36^ for neoantigen-HLA binding or GLIPH^37^ and TCRdist^38^ for neoantigen-TCR recognition—resulting in poor generalizability. In contrast, modern AI-driven tools have achieved pan-antigen and pan-HLA prediction: neoantigen-HLA binding models such as NetMHCPan^32^ (area under the receiver operating characteristic curve, AUROC = 0.92 for MHC-I), NetMHCIIPan,^39^ and HLAIIPred^40^ leverage transformer architectures to encode amino acids as tokens, using attention mechanisms to characterize binding affinity.

Notably, AI models for neoantigen-TCR interaction prediction have advanced substantially, with ERGO2,^41^ ImRex,^42^ and pMTnet^43^ enabling precise TCR recognition forecasting. These tools integrate TCRβ chain CDR3 sequences, complementarity-determining regions (CDRs), and neoantigen structural features. To further enhance accuracy, modern AI platforms adopt hierarchical fusion strategies to integrate epigenomic and proteomic data with genomics, transcriptomics, immunopeptidomics, and immune repertoire sequencing. Epigenomic data refine tumor specificity by identifying epigenetically silenced normal tissue antigens, while proteomic data validate neoantigen presentation on tumor cell surfaces, reducing false positives by 30%.^44^ Graph neural networks (GNNs) further map neoantigen distribution across tumor subpopulations, accounting for intratumoral heterogeneity. Collectively, these innovations have boosted clinically valid neoantigen identification rates from <5% to 32% in trial NCT05721644, with personalized vaccines achieving objective response rates (ORRs) of 46% in melanoma and pancreatic cancer cohorts, underscoring AI’s transformative role in bridging computational prediction and clinical efficacy.^45^ Driven by the continuous development of AI technologies, it is anticipated that the false-positive rate can be maintained at a level below 15% in the future; nevertheless, this prospective target still demands validation from more comprehensive clinical trials.

Despite substantial advancements in *in silico* neoantigen prediction, the accuracy of current tools remains constrained by prevalent false positives and false negatives, which stem from several critical technical and data-related limitations. The root causes of false positives/false negatives in current *in silico* neoantigen prediction tools mainly lie in four aspects. First, most tools rely on sequence-level features rather than atomic-level interactions, failing to capture crucial non-covalent bonds (e.g., hydrogen bonds) that govern T cell activation. Second, incomplete modeling of the HLA-neoantigen-TCR axis—most tools predict only neoantigen-HLA binding or neoantigen-TCR interactions in isolation, neglecting their synergy. Third, the scarcity of high-quality crystal structures of HLA-neoantigen-TCR complexes limits the training of structure-based models. Fourth, independent encoders for neoantigens and HLAs lack adaptability, unable to adjust embeddings dynamically. Additionally, insufficient mono-allelic HLA-II-binding data and sequence similarity biases in training datasets further compromise prediction accuracy, leading to misclassification of immunogenic neoantigens.

## Vector systems of tumor neoantigen vaccines

### Peptide/protein vaccines

Peptide-based cancer vaccines are primarily categorized into short and long peptides. Short peptides (8–10 amino acids), representing minimal T cell epitopes, often bind directly to MHC-I molecules without requiring neoantigen processing by professional APCs, leading to limited immune responses and potential tolerance. In contrast, long peptides (typically >14 amino acids) must be internalized, processed, and presented by APCs, thereby promoting stronger and more sustained T cell activation through co-stimulatory signaling. Antigen insertion and expression control require long-peptide design to include MHC-I and MHC-II restricted epitopes. Chemical modifications such as acetylation and amidation enhance *in vivo* stability. Innate immune characteristics rely on exogenous adjuvants such as Montanide ISA 51 to activate toll-like receptor (TLR) signaling. These peptides lack built-in immune stimulatory modules, resulting in relatively weak immunogenicity. Promising clinical results have been demonstrated with personalized long-peptide vaccines. For instance, NEO-PV-01, a poly-neoantigen vaccine comprising up to 20 patient-specific long peptides, combined with PD-1 inhibition and chemotherapy, showed favorable safety and induced robust T cell responses in advanced cancers, with 55% of vaccine epitopes eliciting immune activation.^46^ Similarly, the NeoVax vaccine, consisting of up to 20 neoantigen peptides, induced persistent and broadening T cell immunity in melanoma patients, with sustained survival benefits observed in early-phase trials.^47^ These findings underscore the potential of long-peptide vaccines in generating durable and specific antitumor immunity.

### Nucleic acid vaccines

These vaccines utilize *in vitro*-transcribed mRNA that encodes tumor-specific or TAAs. The mRNA is delivered via carriers such as lipid nanoparticles (LNPs) to ensure efficient uptake and translation. Antigen insertion and expression control regulate the translation efficiency of neoantigen-encoding mRNA through codon optimization, addition of a 5′ cap structure and a 3′ poly(A) tail. Innate immune characteristics involve mRNA itself exerting adjuvant effects by activating the RIG-I/MAVS pathway, while LNP carriers enhance intracellular delivery efficiency. Yet mRNA is susceptible to nuclease degradation, which compromises expression durability. Once internalized, they elicit potent humoral and T cell-mediated immune responses without genomic integration, offering favorable safety, rapid production, and scalability.^48^ In this review, the application of nucleic acid vectors in neoantigen vaccines serves as the primary focus, while vectors not associated with neoantigens are only incorporated for the purpose of technical comparison.

Promising candidates such as mRNA-4157 (Merck and Moderna), combined with PD-1 blockade, have significantly reduced recurrence and death risks in melanoma in phase IIb trials.^49^ Similarly, BioNTech’s autogene cevumeran, a personalized neoantigen vaccine, has extended recurrence-free survival (RFS) in pancreatic cancer patients when combined with immune checkpoint inhibitors (ICIs) and chemotherapy.^50^ Nevertheless, no mRNA cancer vaccine has yet received clinical approval. However, ongoing optimizations in mRNA structure, stability, and delivery methods, coupled with the inherent advantages of personalized formulation, low manufacturing cost, and rapid scalable production, are poised to unlock their potential as a pivotal strategy in future cancer therapy. A substantial expansion in the clinical testing of mRNA cancer vaccines is anticipated, although widespread clinical approval remains contingent on further validation of efficacy and safety in ongoing trials.

### Cell-based vaccines

As the most potent professional APCs, DCs play a pivotal role in tumor immunotherapy by efficiently capturing, processing, and presenting tumor antigens to activate T cell responses while also stimulating B cells and modulating immune function.^51^ Antigen insertion and expression control involve *ex vivo* pulsing of DCs with neoantigen peptides or transfection via viral vectors to enable DCs to endogenously express neoantigens. Innate immune characteristics include DCs acting as professional APCs that provide co-stimulatory signals via CD80/CD86 and possess inherent immune memory induction capabilities. However, the complex *ex vivo* culture process results in high production costs. A 2021 phase I clinical trial (NCT02956551) evaluating a personalized neoantigen-loaded DC vaccine (Neo-DCVac) in 12 patients with advanced metastatic lung cancer demonstrated promising outcomes. The vaccine exhibited a favorable safety profile, with an ORR of 25%, a disease control rate (DCR) of 75%, a median progression-free survival (PFS) of 5.5 months, and a median overall survival (OS) of 7.9 months. Notably, when combined with ICIs, Neo-DCVac showed synergistic effects, providing early clinical evidence that neoantigen-based DC vaccines can effectively induce specific T cell immunity and exhibit therapeutic potential in cancer patients.^52^ *Ex vivo* activation and antigen loading design of DC vaccines ensure efficient presentation of neoantigens while the complex manufacturing process leads to batch-to-batch variability which may compromise the stability of clinical efficacy.

### Viral vector vaccines

Viral vector vaccines, utilizing replication-deficient or attenuated viruses such as adenovirus, poxvirus, and herpesvirus, represent a promising approach in cancer immunotherapy. Their well-established platform offers a favorable safety profile and the distinct advantage of inducing potent, specific T cell responses without the need for an adjuvant.^53^ A phase I trial evaluated a personalized heterologous vaccine regimen using a chimpanzee adenovirus (ChAd68) primer followed by a self-amplifying mRNA (samRNA) booster, both encoding patient-specific neoantigens. In patients with metastatic microsatellite-stable colorectal cancer, this vaccine successfully induced CD8^+^ T cell responses against predicted neoantigens and was associated with improved OS. Critically, the regimen demonstrated a manageable safety and tolerability profile, with no dose-limiting toxicities observed. Treatment-related adverse events, including fever, fatigue, and transient laboratory abnormalities, were generally acceptable. These findings underscore the potential of advanced viral vector platforms to generate effective, personalized antitumor immunity, marking a substantial step forward in the field.^54^

### Microbial vector vaccine

Microbial vectors have emerged as compelling candidates for neoantigen delivery due to their intrinsic immunogenic properties, which have been rapidly advanced by the development of synthetic biology. Bacterial vector vaccines employ attenuated, non-pathogenic bacteria as live delivery platforms to present heterologous antigens to the host immune system. Their unique mechanism is rooted in their natural biology: after administration, they can invade or be phagocytosed by APCs, particularly DCs and macrophages. Within the phagosome, engineered vectors either secrete or release antigens into the cytosol. For instance, as recently reported in Nature,^26^ engineered *Escherichia coli* Nissle 1917 (*Ec*N) can be systematically optimized through synthetic biology approaches to enhance both neoantigen production efficiency and targeted cytosolic delivery in APCs. They are designed to be more susceptible to blood clearance and phagocytosis, thus improving safety. Microbes naturally home to tumors, such as anaerobic or facultative anaerobic bacteria that can colonize the hypoxic TME. In terms of immune effects, these vectors can trigger potent antitumor immunity. They activate DCs, leading to the extensive priming and activation of neoantigen-specific CD4^+^ and CD8^+^ T cells. There is also broader activation of T and natural killer (NK) cells while reducing tumor-infiltrating immunosuppressive myeloid, regulatory T, and B cell populations. In murine models, such microbial vaccines can effectively control or even eliminate tumor growth and extend survival, demonstrating their potential for precision cancer immunotherapy.

A primary advantage is their intrinsic adjuvant properties. Bacterial cell-wall components and unmethylated CpG DNA are potent pathogen-associated molecular patterns (PAMPs). These molecules engage TLRs and other pattern recognition receptors (PRRs) on APCs. This triggers innate immune activation, inflammasome formation, and pro-inflammatory cytokine production (e.g., IL-12, IL-1β, tumor necrosis factor [TNF]-α), creating a microenvironment that potently drives Th1-type adaptive immunity without need for exogenous adjuvants. Their tumor-targeting potential is significant. Attenuated bacterial vectors exhibit natural tropism for the immunosuppressive TME. Once inside, they can directly infect tumor-associated APCs and even tumor cells themselves, delivering TAAs and simultaneously “re-educating” the TME via PAMP signaling. This reverses local immunosuppression, promotes cytotoxic T lymphocyte infiltration, and can break immune tolerance. Notably, facultative intracellular bacteria such as *Listeria* can escape the phagosome via listeriolysin O, facilitating antigen entry into the host cell cytoplasm. This leads to efficient MHC-I and MHC-II presentation, thereby stimulating robust, antigen-specific CD8^+^ and CD4^+^ T cell responses—a critical advantage for combating intracellular pathogens and cancer.

Bacteria possess inherent tumor tropism predominantly shaped by the TME featuring hypoxia, low pH, and immune suppression. Anaerobic and facultative anaerobic strains, including *E. coli* Nissle 1917,^55^ attenuated *Salmonella typhimurium*,^56^ and *Bifidobacterium*, selectively colonize hypoxic tumor regions via differential growth rather than traditional chemotaxis.^57^ The IL-10 R hysteresis effect—mediated by IL-10/STAT3 positive feedback—enables bacteria to inhibit neutrophil motility for immune evasion in tumors while being cleared in normal tissues.^56^ Genetically engineered strains, such as near-infrared-light-responsive or toxicity-gene-knockout variants, enhance targeting specificity and safety. Beyond colonization, they activate tumor-resident memory CD8^+^ T cells and serve as controllable delivery vehicles for therapeutics. This natural tropism combined with synthetic biology modifications renders bacteria promising tools for targeted therapy of solid tumors such as colorectal cancer.

However, safety and reactogenicity are paramount concerns, as live-attenuated strains carry a risk, however small, of reversion to virulence or causing severe infection in immunocompromised hosts. The strong inflammatory response, while immunostimulatory, can lead to significant transient toxicity. Preexisting immunity against the bacterial vector from prior exposure or vaccination may accelerate vector clearance, reducing vaccine take and efficacy. Technical challenges include maintaining a balance between sufficient attenuation for safety and adequate immunogenicity, ensuring stable plasmid or chromosomal antigen expression *in vivo*, and developing optimal lyophilization processes for oral formulations. Finally, complex manufacturing and regulatory pathways for live biological agents pose significant hurdles for clinical translation.

### Nanovaccine

The application of nanotechnology in neoantigen delivery has significantly advanced cancer immunotherapy. Nanomaterials enhance immunogenic cell death (ICD) through various therapeutic modalities such as phototherapy, radiotherapy, and chemotherapy, promoting the release of endogenous neoantigens.^58^^,^^59^^,^^60^ They efficiently capture and protect these neoantigens from degradation via hydrophobic interactions or functional groups (e.g., maleimide), facilitating their delivery to APCs.^61^^,^^62^ This improves cross-presentation and DC maturation, leading to robust T cell responses.^63^

Self-assembling nanoparticle vaccines (SANVs) have emerged as a promising platform for *in situ* cancer immunotherapy, with unique mechanisms and distinct advantages. Their core mechanism relies on the spontaneous self-assembly of building blocks. This assembly is driven by hydrophobic interactions, electrostatic forces, or hydrogen bonding, forming well-defined nanostructures (10–200 nm) that mimic pathogen sizes. This structural feature enables passive targeting to lymph nodes through the enhanced permeability and retention effect, prolonging antigen retention by 5- to 10-fold and facilitating efficient uptake by APCs. Moreover, nanomaterials prolong neoantigen retention in the TME through the enhanced permeability and retention effect, sustaining immune activation.^62^ Representative studies include programmable DNA nanoclusters for ICD induction^60^ and PBA-nChap for neoantigen stabilization.^61^ These innovations highlight the potential of nanotechnology in optimizing neoantigen-based immunotherapies.

**Figure 2:**
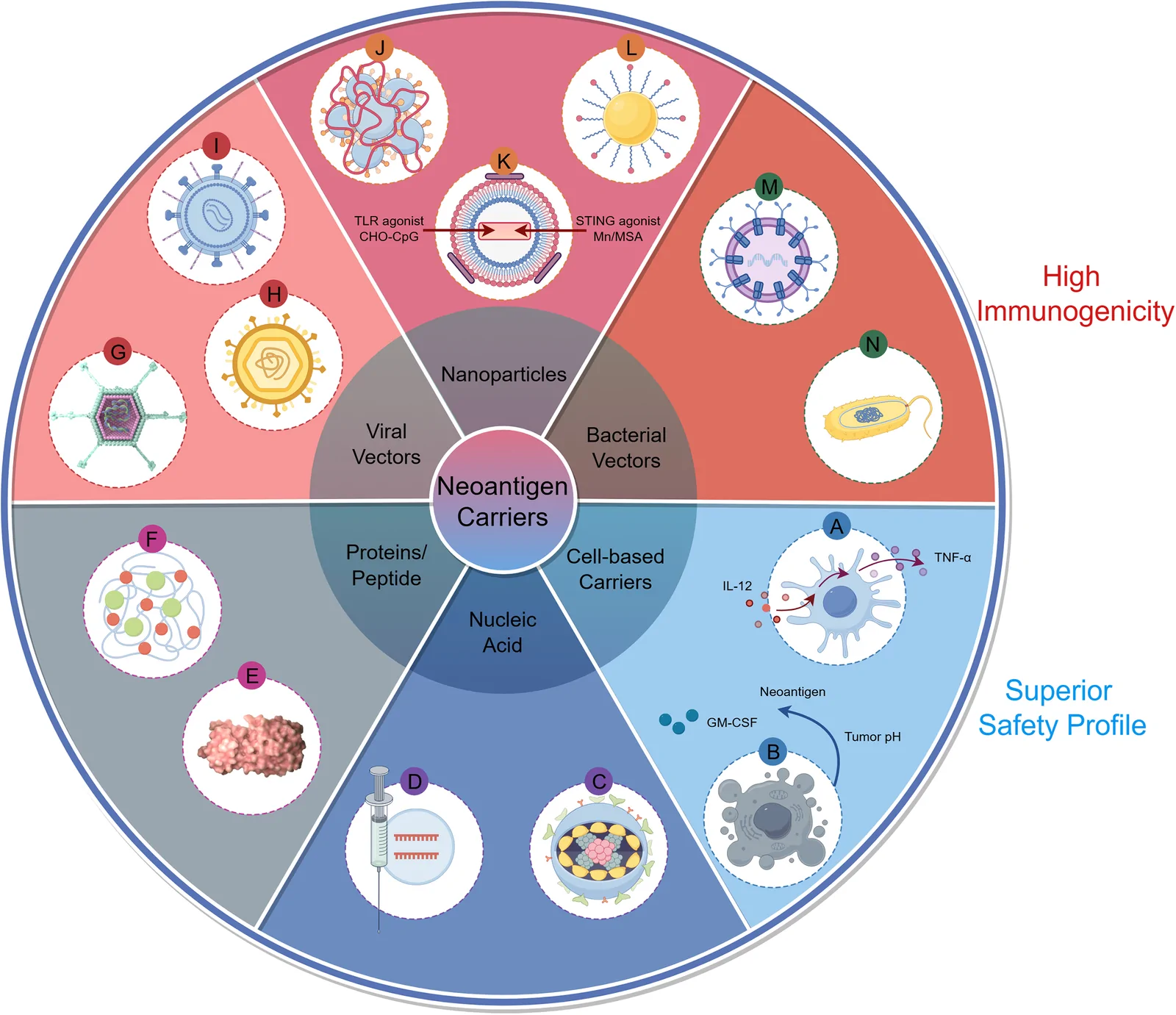
Schematic overview of major neoantigen vaccine vector platforms (A) dendritic cell (DC) vaccines (e.g., autologous DCs pulsed with tumor neoantigens); (B) whole tumor cell vaccines (irradiated autologous/allogeneic tumor cells) and genetically modified tumor cells (e.g., expressing granulocyte-macrophage colony-stimulating factor (GM-CSF) or other immunostimulatory molecules); (C) DNA vaccines (plasmid DNA, often delivered via electroporation or nanoparticles); (D) mRNA vaccines (delivered via lipid nanoparticles [LNPs]); (E) recombinant protein vaccines (e.g., fusion proteins with immune stimulators); (F) synthetic peptide vaccines; (G) adeno-associated virus (AAV); (H) herpes simplex virus; (I) lentivirus; (J) polymeric nanoparticles (e.g., PLGA-poly(lactic-co-glycolic acid)); (K) liposomes (e.g., cationic liposomes for nucleic acid delivery); (L) gold nanoparticles (AuNPs; used for neoantigen conjugation); (M) outer-membrane vesicles (OMVs); (N) attenuated bacteria. The blue-labeled platforms (cell-based vaccines, whole-nucleic-acid vaccines, and peptide/protein vaccines) exhibit superior safety and established manufacturing, although their efficacy is often constrained by the tumor immune microenvironment. The red-labeled platforms (viral vectors, nanoparticles, bacterial vectors) are emerging as promising candidates with high immunogenicity, but large-scale clinical trials remain limited. In this review, the vectors discussed herein are centered on their applications in neoantigen vaccines, and applications in TAAs are incorporated solely for the purpose of technical comparison.

A key advantage is their intrinsic adjuvant properties—many SANVs eliminate the need for exogenous adjuvants. For example, polyhydrophobic amino acid-based nanofibers activate APCs via membrane interactions, while virus-like particles (VLPs) trigger PRRs to induce robust immune responses.^64^ SANVs also support modular multi-epitope loading, covering tumor heterogeneity, and are biocompatible due to biodegradable materials, minimizing long-term toxicity.^65^ Active targeting can be achieved by modifying surfaces with ligands such as RGD peptides, enhancing tumor-specific accumulation.^66^ However, large-scale manufacturing faces challenges in maintaining size uniformity and epitope display consistency. Their size (>50 nm) restricts penetration into solid tumor cores, especially in desmoplastic tumors.^67^ Currently, most SANVs are in preclinical or early phase I trials, lacking long-term efficacy and safety data. Future efforts should focus on optimizing scalable production, improving tumor penetration via surface modification, and validating clinical utility in combination with immunotherapies.^68^ Each of the aforementioned vector platforms possesses inherent advantages and limitations (Figure 2 and Table 2). Their clinical translation efficacy depends not only on the intrinsic design of the vectors themselves but also on the accuracy of neoantigen screening and the rational stratification strategies for patient populations. In the following sections, we focus on the clinical research progress and core insights of neoantigen vaccines.

**Table 2:** Comparison of diverse neoantigen vaccine platforms for cancer immunotherapy

Vaccine platform	Mechanism	Advantages	Key limitations
Synthetic peptide vaccines	short peptides bind directly to MHC class I molecules; long peptides are processed and presented by APCs	(1) high safety profile (no genomic integration risk, mild AEs); (2) flexible personalized design (targeted neoantigen epitope screening); (3) mature manufacturing process	(1) weak immunogenicity (exogenous adjuvant-dependent); (2) poor *in vivo* stability (rapid protease degradation); (3) efficacy limited by immunosuppressive TME; (4) synthetic bottlenecks for long peptides (low purity, high cost)
mRNA vaccines	*in vitro*-transcribed mRNA is delivered *in vivo* to translate neoantigens, activating humoral and cellular immunity	(1) rapid production cycle; (2) comprehensive immune response; (3) scalable manufacturing (cost-effective, no cell culture required)	(1) intrinsic instability; (2) weak expression persistence; (3) efficacy limited by immunosuppressive TME
DC vaccines	DC cells are activated and loaded with neoantigens *ex vivo*, then reinfused to efficiently present antigens to T/B cells and regulate the immune microenvironment	(1) excellent safety profile (autologous origin, no severe AEs); (2) induces long-term immune memory (sustained antitumor protection); (3) efficient antigen presentation	(1) complex manufacturing process (*ex vivo* cell culture + neoantigen loading, 2–4 weeks); (2) high production cost; (3) efficacy limited by immunosuppressive TME; (4) low patient compliance
SANVs	form nanostructures (10–200 nm) via hydrophobic/electrostatic self-assembly, targeting lymphoid organs and sustaining neoantigen release	(1) intrinsic adjuvant properties (no exogenous adjuvants needed); (2) strong lymphoid targeting; (3) good biocompatibility; (4) multi-epitope loading (covers tumor heterogeneity)	(1) high difficulty in large-scale production (batch-to-batch variability); (2) limited penetration of solid tumors (structures >50 nm hard to enter tumor cores); (3) sparse clinical data
Engineered microbial vectors	anaerobic/facultative anaerobic microbes (bacteria/phages) actively target hypoxic TME, colonize, and release neoantigens while activating innate immunity	(1) active tumor targeting (enrichment in hypoxic regions); (2) dual immunomodulation (neoantigen presentation + TME improvement); (3) low production cost (large-scale cultivable); (4) deep tumor penetration	(1) vector clearance by immune system; (2) toxicity risks (high doses may induce inflammation); (3) insufficient clinical validation

This table primarily focuses on the application of vectors in neoantigen vaccines, while non-neoantigen vectors are included solely for technical comparison and reference.

## Clinical progress of cancer neoantigen vaccines

Neoantigen vaccines have emerged as a promising form of personalized immunotherapy for solid tumors. Several clinical trials have already reported results in malignancies such as melanoma, glioblastoma (GBM), gastrointestinal cancers (including gastric and colorectal cancer), hepatocellular carcinoma, and pancreatic cancer, demonstrating favorable safety profiles and encouraging efficacy, particularly when combined with ICIs.

Building on these findings, numerous trials are actively investigating this approach. Table 3 summarizes the key characteristics of these ongoing clinical trials, including vaccine platforms, combination regimens, and phase distribution. The following sections first review the advances based on reported outcomes according to cancer type.

**Table 3:** Ongoing clinical trials of neoantigen vaccines in human malignancies and early results

NCT number	Antigen type	Vector	Combination therapy	Cancer type	Phase	Institution	Early results (reported data)
NCT05916248	personalized neoantigen	mRNA vaccine (mRNA-0217)	PD-1 inhibitor (pembrolizumab)	advanced solid tumor	early phase Ⅰ	Ruijin Hospital	not reported
NCT06141369	personalized neoantigen	mRNA vaccine (mRNA-0523-L001)	N/A	advanced endocrine tumor	–	Shanghai Jiao Tong University School of Medicine	not reported
NCT06682117	personalized neoantigen	DC vaccine (Neo-DCVac)	–	advanced solid tumors	N/A	Fudan University	not reported
NCT06751849	personalized neoantigen	DC vaccine	PD-1 inhibitor and radiotherapy	advanced NSCLC	N/A	The First Affiliated Hospital of Nanchang University	not reported
NCT06675201	personalized neoantigen	DC vaccine (Neo-DCVac)	radical chemoradiotherapy or chemoradiation-immunotherapy	esophageal squamous cell carcinoma	phase Ⅱ	Sichuan University	not reported
NCT04147078	personalized neoantigen	DC vaccine	N/A	postoperative locally advanced gastric cancer, hepatocellular carcinoma, lung cancer, and colorectal cancer	phase Ⅱ	Sichuan University	ASCO 2023: *n* = 13, 1-year RFS: 83% (postoperative gastric cancer/hepatocellular carcinoma/lung cancer/colorectal cancer), 5/13 evaluable patients developed specific T cell responses to >50% of vaccine neoantigens; median RFS/OS not reached
NCT05743595	personalized neoantigen	DC vaccine	PD-1 inhibitor (retifanlimab)	newly diagnosed, unmethylated glioblastoma	phase Ⅰ	Washington University School of Medicine	not reported
NCT06314087	personalized neoantigen	peptide vaccine (iNATURE)	radiotherapy	advanced malignant solid	phase Ⅰ	The University of Hong Kong-Shenzhen Hospital	not reported
NCT05269381	personalized neoantigen	peptide vaccine	PD-1 inhibitor (pembrolizumab)	advanced solid tumors	phase Ⅱ	Mayo Clinic	not reported
NCT05013216	neoantigen KRAS	long-peptide vaccine	N/A	high-risk pancreatic cancer	phase Ⅰ/Ⅱ	Sidney Kimmel Comprehensive Cancer Center at Johns Hopkins et al.	AACR 2024: *n* = 15, All 15 patients (100%) developed mKRAS-specific T cell responses
NCT05098210	personalized neoantigen	peptide vaccine	N/A	stage IIIC-IV melanoma, hormone receptor-positive HER2-negative metastatic refractory breast cancer or stage III–IV non-small cell lung cancer	phase Ⅰ	Fred Hutchinson Cancer Center	not reported
NCT06529822	personalized neoantigen	long-peptide vaccine	N/A	muscle-invasive bladder carcinoma	phase Ⅰ	Washington University School of Medicine	not reported
NCT03606967	personalized neoantigen	long-peptide vaccine	PD-L1 inhibitor (durvalumab) and CTLA-4 inhibitor (tremelimumab) and chemotherapy	metastatic triple-negative breast cancer	phase Ⅰ	National Cancer Institute	not reported
NCT04248569	neoantigen DNAJB1-PRKACA fusion kinase	peptide vaccine	PD-1 inhibitor (nivolumab) and CTLA-4 inhibitor(ipilimumab)	fibrolamellar hepatocellular carcinoma	phase Ⅱ	Sidney Kimmel Comprehensive Cancer Center at Johns Hopkins	Nature Medicine 2025: 16 patients enrolled, 12 patients completed the priming phase and were evaluable ORR 25% (3/12); DCR: 75% (9/12) in fibrolamellar hepatocellular carcinoma patients (metastatic setting)^69^
NCT04930783	personalized neoantigen	peptide vaccine	CDX-301 and PD-1 inhibitor (nivolumab or pembrolizumab)	melanoma	phase Ⅰ	Dana-Farber Cancer Institute	not reported
NCT02287428	personalized neoantigen	peptide vaccine	PD-1 inhibitor (pembrolizumab) and radiotherapy	newly diagnosed glioblastoma	phase Ⅰ	Dana-Farber Cancer Institute	ASCO 2021: *n* = 12, (9 radiographically evaluable patients) 6 patients SD, 3 patients PD
NCT03219450	personalized neoantigen vaccine	peptide vaccine	anti-PD-1 (pembrolizumab) and chemotherapy (cyclophosphamide)	lymphocytic leukemia	phase Ⅰ	Dana-Farber Cancer Institute	not reported
NCT04943848	neoantigen rHSC-DIPGVax, off-the-shelf neoantigen heat shock protein containing 16 peptides	off-the-shelf neoantigen peptide vaccine	PD-1 inhibitor (balstilimab) and CTLA-4 inhibitor (zalifrelimab)	diffuse intrinsic pontine glioma and diffuse midline glioma, with H3 K27M-mutant	phase Ⅰ	Ann & Robert H Lurie Children’s Hospital of Chicago	not reported
NCT06344156	neoantigen	mRNA vaccine	anti-PD-1 and chemotherapy	pancreatic cancer	phase Ⅰ	Sichuan University	not reported
NCT03897881	personalized neoantigen	mRNA vaccines(mRNA-4157)	anti-PD-1 (pembrolizumab)	high-risk melanoma	phase Ⅱ	Laura and Isaac Perlmutter Cancer Center	Lancet 2024: mRNA-4157 plus pembrolizumab (*n* = 107) vs. pembrolizumab (*n* = 50); 18-month RFS 79% vs. 62%^45^

Data were retrieved from ClinicalTrials.gov.

RFS, recurrence-free survival; SD, stable disease; PD, progressive disease; ORR, objective response rate; DCR, disease control rate; N/A, not available.

### Tumors of the digestive system

Pancreatic ductal adenocarcinoma (PDAC) remains one of the most lethal and therapeutically challenging malignancies due to its aggressive biology and low mutational burden. Two seminal back-to-back studies published in *Nature* highlight innovative immunotherapeutic strategies targeting neoantigens to address this unmet need.^7^^,^^70^ Balachandran et al. report results from a phase I trial using an individualized mRNA-neoantigen vaccine (autogene cevumeran) in combination with atezolizumab (anti-PD-L1) and chemotherapy in surgically resected PDAC patients. The study demonstrated that vaccine responders (eight of 16 patients) exhibited significantly prolonged RFS (primary endpoint; median not reached at 3.2 years) in surgically resected PDAC patients compared to non-responders (median RFS 13.4 months; *p* = 0.007), indicating that robust and durable vaccine-induced CD8^+^ T cell responses can confer potent clinical benefits.^7^ Complementing these findings, a parallel study led by Swanton, McGranahan, and Quezada elucidates how clonal driver neoantigens may be lost under selective pressures from targeted therapy and immune surveillance, underscoring the importance of targeting stable neoantigen pools.^70^ Together, these works provide compelling clinical and mechanistic evidence that personalized RNA neoantigen vaccines can induce long-lived antitumor immunity and improve outcomes in high-risk PDAC, offering a promising strategic direction for adjuvant immunotherapy in solid tumors with historically poor prognosis.

Although neoantigen vaccines have shown impressive clinical results in tumors of the digestive system, the personalized vaccine strategy faces significant challenges due to its reliance on individual tumor mutational profiling and custom manufacturing, which result in extended production times and high costs.^8^ In contrast, off-the-shelf vaccines such as ELI 002, which targets recurrent KRAS mutations including G12D and G12R, offer a more practical alternative. These off-the-shelf vaccines can be produced in bulk at approximately one-third of the cost of personalized versions and are available for immediate use. This approach not only reduces financial barriers but also enables timely treatment initiation, which is critical for preventing relapse in high-risk patients. Emerging clinical evidence indicates that such premanufactured neoantigen vaccines can elicit robust T cell responses and promising survival outcomes, highlighting their potential to broaden access to effective immunotherapy in digestive oncology.^71^

The team at Nanjing Drum Tower Hospital developed a personalized neoantigen nanovaccine (PNVAC) that enhances antitumor immunity and reduces recurrence. In murine models, PNVAC combined with anti-PD-1 significantly improved survival and protection compared to free neoantigens. A phase I clinical trial (ChiCTR1800017319) in patients with resected high-risk gastric/gastroesophageal junction cancer demonstrated promising safety and immunogenicity. The vaccine markedly increased 1- and 2-year disease-free survival rates beyond historical controls. PNVAC induced robust CD4^+^ and CD8^+^ T cell activation, promoted a memory T cell phenotype, and sustained immune responses for over 1 year post-vaccination, offering a feasible strategy to prevent recurrence in postoperative gastric cancer.^58^

### Tumors of the urinary system

Based on a phase I trial (NCT02950766) in high-risk, resected clear cell renal cell carcinoma (RCC),^72^ personalized cancer vaccines (PCVs) targeting neoantigens demonstrate highly promising efficacy and immunogenicity. At a median follow-up of 40.2 months, none of the nine vaccinated patients experienced disease recurrence. The vaccine was well tolerated with no dose-limiting toxicities. Critically, all patients developed T cell responses against vaccine antigens, including mutations in key RCC driver genes such as VHL, PBRM1, and BAP1. A durable expansion of peripheral T cell clones was observed post vaccination, and T cell reactivity against autologous tumor cells was detected in seven of nine patients. These results indicate that PCVs can elicit potent and targeted antitumor immunity, supporting their potential as an effective adjuvant therapy for RCC, even in a tumor type with typically low mutational burden.

### Tumors of the respiratory system

Lung cancer, which has the highest global incidence and mortality among all malignancies, presents limited treatment options for advanced patients who have failed first-line therapies. Given its high tumor mutational burden and abundant neoantigen load, non-small cell lung cancer (NSCLC) represents a promising target for neoantigen-based vaccination. A phase Ⅰ trial by The University of Texas MD Anderson Cancer Center evaluated personalized neoantigen vaccination (PPV) in advanced NSCLC.^73^ The treatment was safe and feasible. Strikingly, all seven clinical responders had EGFR mutations and demonstrated T cell immunity against shared EGFR neoantigens (L858R/T790M). This study establishes these EGFR variants as potent, shared immunotherapeutic targets and suggests that combining PPV with EGFR TKIs is a promising strategy for this patient population. A phase Ⅰ investigator-initiated trial at West China Hospital evaluated a personalized neoantigen DC vaccine in 12 heavily pretreated patients with metastatic lung cancer. The vaccine, featuring 12–30 patient-specific peptides, was safe with only grade 1–2 adverse events. Clinical outcomes showed an ORR (secondary endpoint) of 25%, DCR of 75%, median PFS of 5.5 months, and median OS of 7.9 months in heavily pretreated metastatic lung cancer patients. This pilot study demonstrates the feasibility, safety, and promising antitumor activity of a personalized neoantigen DC vaccine, offering a novel therapeutic strategy for advanced lung cancer.

### Head and neck tumors

Neoantigen vaccines represent a promising therapeutic approach for head and neck squamous cell carcinoma (HNSCC), particularly in HPV-positive tumors where viral oncoproteins (E6/E7) serve as immunogenic targets, as well as in HPV-negative tumors with high mutational burden. The presence of tumor-infiltrating lymphocytes in HNSCC suggests an immune-responsive microenvironment, supporting the rationale for vaccine-based strategies. However, challenges remain, including overcoming T cell dysfunction in the TME and identifying immunogenic neoantigens with high affinity while avoiding cross-reactivity with self-proteins. Current efforts focus on enhancing vaccine efficacy through combination with ICIs, leveraging synergistic mechanisms to boost antitumor immunity.

Several clinical trials are evaluating neoantigen vaccines in combination with ICIs across different stages of development. Examples include peptide-based vaccines such as ISA101 with nivolumab (NCT03669718, phase Ⅱ), DNA vaccines such as MEDI0457 (phase Ⅰ/Ⅱ), and novel approaches such as the liposomal HPV16 E6/E7 vaccine PDS0101 combined with pembrolizumab (NCT04260126, phase Ⅱ). Additionally, viral vector vaccines (e.g., axalimogene filolisbac) and engineered fusion proteins (HPV16 E7-pHLA-IL2-Fc + pembrolizumab, NCT03978689) are being tested, with early data showing T cell clonotype expansion. These trials aim to address key limitations in vaccine monotherapy while exploring optimal combinations to improve clinical outcomes in HNSCC.

### Tumors of the nervous system

GBM remains an incurable primary brain tumor with dismal prognosis. Standard of care, including radiotherapy with concurrent and adjuvant temozolomide, yields a median OS of merely 15 months^74^ Subsequent US Food and Drug Administration (FDA) approvals of bevacizumab for recurrent GBM and tumor-treating fields for both newly diagnosed and relapsed GBM demonstrated limited clinical benefit, improving PFS without impacting OS.^75^ Despite therapeutic advances, tumor recurrence is inevitable, underscoring the critical need for novel treatment paradigms to overcome therapeutic resistance in GBM. The CNS maintains an immune-privileged status due to the blood-brain barrier (BBB), which restricts immune cell trafficking, and its unique immunosuppressive microenvironment featuring low MHC expression, limited antigen presentation, and dominant regulatory T cell (Treg) activity. Tumor-associated microglia and astrocytes further contribute to immune evasion.^76^

Personalized neoantigen vaccines represent a breakthrough approach for GBM, demonstrating superior potential compared to conventional tumor-associated antigen vaccines. Recent clinical data from 173 GBM patients treated with individualized neoantigen peptide vaccines show promising results: median OS reached 31.9 months from diagnosis, with 90% of monitored patients developing vaccine-specific T cell responses.^77^ Notably, patients exhibiting robust multi-peptide immune responses achieved significantly longer survival (53 vs. 27 months, *p* = 0.03). The treatment maintains an excellent safety profile, with predominantly grade 1–2 adverse events. While these results from compassionate use demonstrate feasibility and immunogenicity, they also highlight the critical importance of inducing broad, durable T cell responses. The observed survival extension, particularly in responders, suggests neoantigen vaccination may help overcome GBM’s immunosuppressive microenvironment, although randomized controlled trials are needed to confirm efficacy compared to standard care.

### Experience and insights from successful neoantigen-based clinical trials

In recent years, neoantigen-based clinical trials have been continuously updated, with both successes and failures. Through analysis, we found that most successful neoantigen-based clinical trials share the following four characteristics. Firstly, strict antigen screening ensures efficacy: for example, a personalized RNA neoantigen vaccine reported by Rojas et al. underwent rigorous neoantigen screening via whole-exome sequencing and RNA-seq, combined with HLA-binding-affinity prediction and immunogenicity verification, to select tumor-specific neoantigens with high presentation potential. In a phase Ⅱ trial for resected pancreatic cancer, this strict screening strategy contributed to a 60% patient response rate. Vaccine-responsive patients showed significantly expanded neoantigen-specific CD8^+^ T cell clones and a 72% reduction in recurrence risk compared to non-responders (hazard ratio [HR] = 0.28, 95% confidence interval [CI] 0.09–0.87, *p* = 0.026).^50^ Secondly, multi-epitope design covers tumor heterogeneity, as exemplified by the personalized mRNA vaccine mRNA-4157 (V940) in the KEYNOTE-942 trial: combined with pembrolizumab, it reduced the risk of distant metastasis or death by 62% and the risk of recurrence or death by 49% in high-risk melanoma patients, with the 18-month distant metastasis-free survival rate reaching 91.8%.^45^ Thirdly, combination with ICIs reverses T cell exhaustion: a personalized neoantigen-loaded DC vaccine plus ICI induced durable complete regression (over 25 months) in metastatic gastric cancer, with neoantigen-specific T cell clones significantly expanded.^78^ Fourthly, rational patient stratification (high TMB, abundant immune infiltration) improves response, as seen in neoantigen vaccine trials for melanoma: Wells et al. identified TMB as a pivotal biomarker, noting high TMB correlates with increased neoantigen load and robust immunity, and stratification by TMB elevated vaccine response rates.^34^^,^^79^ These four core characteristics, strict antigen screening, multi-epitope design, ICI combination, and rational patient stratification, synergistically enhance the efficacy of neoantigen-based immunotherapy, and their validation in multiple trials provides key references for the clinical translation and scheme optimization of personalized cancer immunotherapy.

### Lessons learned from unsuccessful neoantigen-based clinical trials

While encouraging clinical responses have been reported, not all patients benefit from neoantigen-based therapies. Several high-profile failures further highlight core bottlenecks, which can be summarized as follows: Firstly, antigenic loss and immune-hostile microenvironment lead to treatment failure, exemplified by the personalized neopeptide vaccine targeting EGFR ex19del,^70^ which elicited systemic T cell reactivity but failed to control tumor progression. One of primary causes is antigenic loss via chromosomal instability, especially in post-whole-genome-doubling mutations. Secondly, prediction-T cell repertoire limitations pose critical barriers: even in melanomas with high neoantigen load, the actual range of neoantigens recognized by T cells is far narrower than predicted, and this recognition correlates with clinical responses. This directly confirms a substantial gap between predicted and actual immunogenicity, and the inherent limitations of the T cell repertoire itself further restrict therapeutic efficacy.^80^ Thirdly, in the aforementioned EGFR ex19del-targeted personalized neopeptide vaccine trial, the discrepancy between induced systemic T cell reactivity and lack of tumor control was attributed to an immune-hostile TME characterized by poor T cell infiltration and elevated M2 macrophage polarization—underscoring that vaccine-induced T cell responses alone do not guarantee meaningful clinical benefit.^70^ These three challenges represent critical hurdles that should be addressed for the success of clinical trials of neoantigen vaccines, and the specific mechanisms underlying these issues as well as corresponding solutions are elaborated in detail in the subsequent sections. Successes and challenges in clinical research have revealed the core bottlenecks of neoantigen vaccines in practical applications. These bottlenecks essentially stem from the biological characteristics of neoantigens themselves, the technical limitations of vaccine design, and the complexity of TME. In the next section, we systematically analyze these unmet needs and key limitations.

## Limitations and unmet needs of cancer neoantigen vaccines

### Neoantigen heterogeneity and immune escape

**Figure 3:**
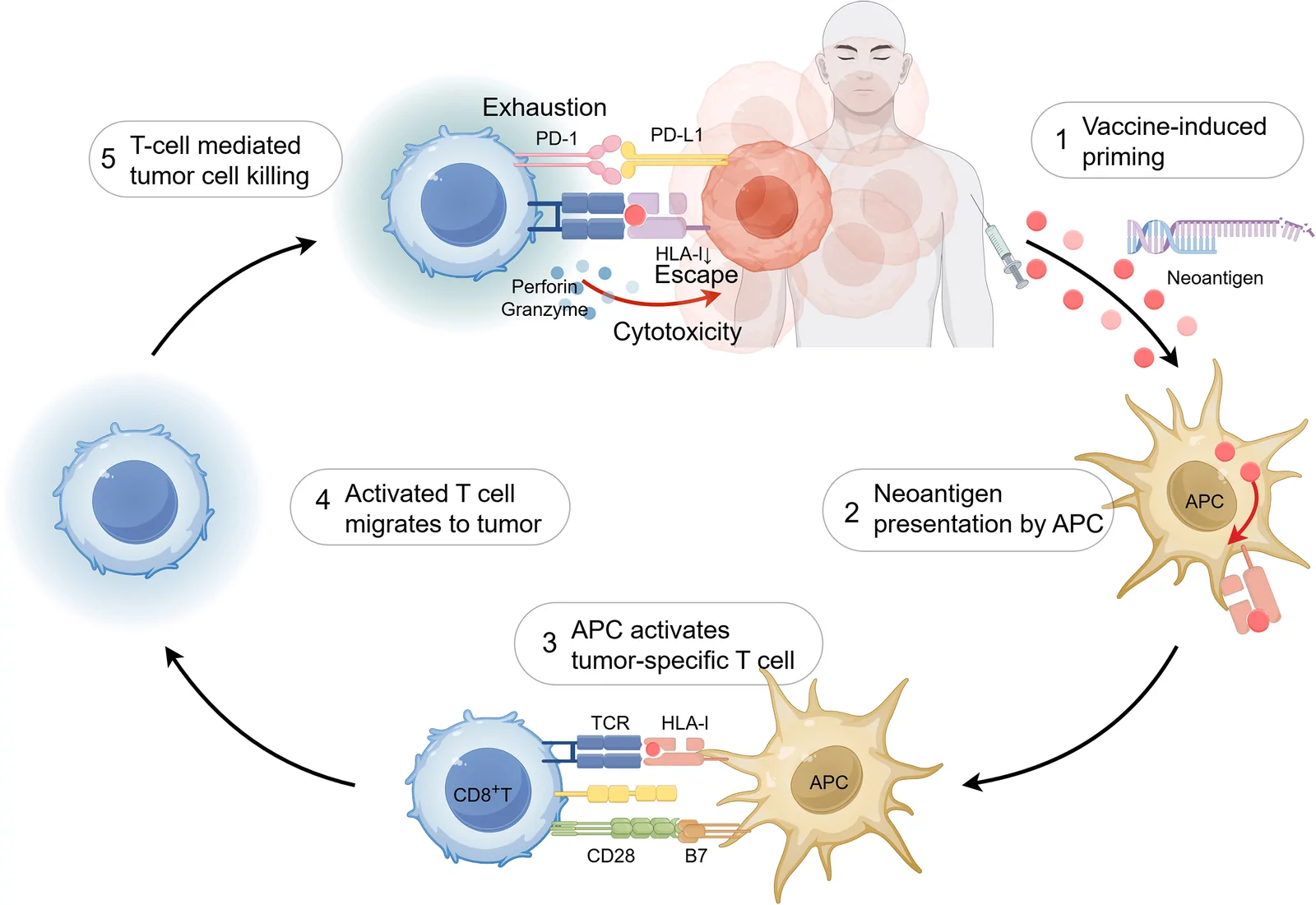
Mechanism of neoantigen vaccine-induced antitumor immunity This diagram illustrates the neoantigen-specific immune activation cascade, from tumor somatic mutation to effector T cell-mediated tumor clearance, with key molecules and bottleneck nodes annotated. (1) Vaccine priming: tumor-specific neoantigens (derived from tumor somatic mutations) are delivered by neoantigen vaccines to initiate immune responses. (2) Neoantigen processing/presentation: antigen-presenting cell (APC) uptake, process neoantigens, and assemble them with HLA-I molecules into surface-presented complexes. (3) Neoantigen-specific CD8^+^ T cell activation: in lymph nodes, APCs activate CD8^+^ T cells via dual signals (neoantigen/HLA-I-TCR binding + B7-CD28 co-stimulation). (4) T cell trafficking: activated cytotoxic T lymphocytes (CTLs) migrate to and infiltrate the TME. (5) Tumor cell killing and bottlenecks: CTLs recognize tumor cells via neoantigen/HLA-I complexes and release perforin/granzyme to induce lysis; key bottlenecks include HLA-I downregulation (HLA-I escape) and T cell exhaustion via PD-1/PD-L1 binding (core targets for immunotherapy optimization). For detailed immune activation mechanisms, refer to core mechanisms of neoantigen-mediated immune activation.

A central challenge in the development of neoantigen-based vaccines stems from the profound spatial and temporal heterogeneity of the neoantigen landscape, which is continuously reshaped by tumor evolution under selective pressures from the immune system and therapeutic interventions. The mechanism is illustrated in Figure 3. While primary tumors frequently harbor clonal mutations shared across most tumor cells, metastatic lesions and residual disease following treatment often display markedly divergent molecular and immunogenic profiles. This divergence is a consequence of ongoing immunoediting and clonal selection. Notably, immunodominant neoantigens—those most capable of eliciting robust and protective T cell responses—are paradoxically the most vulnerable to elimination through a spectrum of immune evasion strategies. Tumors may execute the genomic deletion or epigenetic silencing of genes encoding these potent neoantigens. Furthermore, loss of heterozygosity at HLA class I loci can drastically reduce the diversity of presented peptides. Additionally, tumors frequently downregulate or functionally impair the antigen processing and presentation machinery via mechanisms such as mutations in beta-2-microglobulin or critical components of the peptide-loading complex, including transporters associated with antigen processing or tapasin. These adaptive changes collectively enable tumors to evade T cell recognition even when a response is initially mounted. Therefore, a neoantigen vaccine’s clinical success is critically threatened if its design is primarily anchored to antigens that are subsequently lost or rendered invisible by these escape mechanisms in evolving, resistant, or metastatic subclones. This underscores the necessity for vaccination strategies that target a broader array of clonally stable neoantigens or are combined with interventions that prevent or counteract these adaptive immune escape pathways.^81^

### Predicting neoantigen immunogenicity

Accurate prediction of neoantigen immunogenicity remains a central challenge in vaccine development, heavily constrained by limitations in data availability and quality. Current algorithms rely on often heterogeneous and poorly harmonized datasets, which lack standardized protocols for sequencing, mutation calling, neoantigen prioritization, and immunogenicity validation. Immunogenicity datasets are frequently biased, as they predominantly include pre-selected, high-confidence candidates and omit negative results, thereby skewing model training and validation.

Substantial variability in T cell assays—such as enzyme-linked immunospot assay (ELISpot), intracellular cytokine staining, or MHC multimers—further complicates data integration. These methods differ in sensitivity, target cell populations (CD4^+^ vs. CD8^+^ T cells), and experimental context, leading to inconsistent classification of immunogenic neoantigens. Critical biological and technical metadata are often missing, hampering cross-study comparisons and meta-analyses.

Compounding these issues, the extreme diversity of MHC molecules and T cell repertoires across patients demands very large, well-annotated datasets—far beyond the few hundred samples currently available. Initiatives such as the TESLA consortium represent important efforts toward standardization, but computational innovations—such as active learning and transfer learning—are urgently needed to build robust immunogenicity models from limited data.

### Vaccine design

The design and implementation of neoantigen-based cancer vaccines are fraught with significant challenges that span biological selection, technological platform optimization, and manufacturing scalability. The initial hurdle lies in the selection of neoepitope candidates, which balance inclusion of sufficient mutations (from two to dozens per vaccine) with strategic combination of complementary categories—such as MHC-I and MHC-II restricted epitopes or clonal and subclonal antigens—to mitigate the risk of relying on any single, potentially incorrect, biological hypothesis.

A second layer of complexity arises from the choice of vaccine technology platform—including synthetic long peptides, mRNA, DNA, or viral vectors—all of which remain largely experimental. Each platform necessitates individualized optimization of adjuvants and vaccination schedules, factors that critically influence whether a neoepitope is effectively delivered and its immunogenic potential realized. This variability complicates comparative evaluation and clinical learning.

However, the most formidable obstacle to clinical translation is manufacturing. Producing individualized vaccines necessitates a paradigm shift from traditional bulk drug manufacturing to highly parallelized, small-scale production campaigns, each yielding a unique drug product for a single patient. The viability of this approach hinges on the development of innovative, scalable, and cost-effective manufacturing processes. Future success will likely depend on emerging technologies such as fully digitized and autonomous cloud-controlled production systems, leveraging advances in robotics and miniaturization to make rapid, good manufacturing practice (GMP)-compliant production a reality. To address the three core limitations mentioned above, targeted optimization is required from three dimensions: prediction technologies, efficacy enhancement, and delivery systems. Below, specific strategies and interdisciplinary solutions are elaborated in detail.

## Optimization strategies for cancer neoantigen vaccines

### Advancing neoantigen prediction

As the foundational step for personalized vaccine design, neoantigen prediction directly addresses the core limitation of immune escape driven by intratumoral heterogeneity (see section neoantigen heterogeneity and immune escape). This section outlines integrated computational and experimental strategies to enhance the accuracy, specificity, and comprehensiveness of neoantigen identification. Improved precision enables the detection of a broader, clonally representative set of tumor-specific neoantigens, thereby reducing the risk of escape associated with single-target reliance. Integrating longitudinal multi-regional sampling (including liquid biopsy) captures the spatiotemporal dynamics of neoantigen expression, ensuring the identified panel encompasses both clonal and subclonal alterations. This comprehensive repertoire is critical for establishing robust immune coverage and improving the durability of vaccine-induced antitumor responses. The evolution of neoantigen prediction has been marked by a significant sophistication in computational models. Early tools primarily focused on predicting MHC-binding affinity, a foundational but limited step. The current generation of algorithms has transitioned to integrated frameworks that incorporate multidimensional features of antigen presentation. These advanced models simultaneously evaluate proteasomal cleavage, TAP transport efficiency, and TCR recognition probability alongside MHC binding. This holistic approach more accurately mirrors the biological complexity of the antigen processing and presentation pathway, leading to a substantial improvement in prediction precision and a higher positive predictive value for identifying true immunogenic neoantigens. Xin et al. developed a deep-learning-based neoantigen prediction model, NUCC, which demonstrated superior accuracy in identifying immunogenic epitopes, thereby facilitating the optimization of personalized nanovaccines for gastric cancer therapy.^82^

A critical strategy for enhancing prediction specificity lies in the integrative analysis of multi-omics data. Moving beyond sole reliance on genomic data, the field now leverages transcriptomics to filter for neoantigens derived from actively transcribed genes. Most significantly, the incorporation of immunopeptidomics data—which directly identify peptides presented on the cell surface by MHC molecules—provides a gold standard for both training and validating computational models. This synergy between genomic, transcriptomic, and direct mass spectrometry-based peptidomic data ensures predictions are biased toward neoantigens that are not only generated but also successfully presented on the tumor cell surface.

A crucial paradigm shift is underway, transitioning from predicting MHC binding alone to forecasting functional T cell activation. High MHC-binding affinity is widely recognized as necessary but insufficient for immunogenicity; the contemporary focus is on prioritizing peptides that elicit robust T cell reactivity. This requires training more complex machine-learning models on large-scale datasets generated from *in vitro* functional assays that measure T cell reactivity. By learning the patterns that determine TCR engagement and immune activation, these next-generation models aim to prioritize the most therapeutically relevant neoantigens. Gfeller et al. developed PRIME, a model predicting immunogenic epitopes by integrating antigen-presentation and TCR-recognition mechanisms, validated experimentally and through immunoediting analysis.^36^^,^^83^ Li et al. developed Splicing Neoantigen Finder (SNAF), an AI-driven workflow integrating DeepImmuno (a deep-learning model for immunogenicity prediction), BayesTS (Bayesian tumor-specific scoring), and RNA-SPRINT (RNA splicing regulation analysis). SNAF systematically mines splice-derived neoantigens from RNA-seq data, identifying shared targets across 90% of melanoma patients—overcoming the heterogeneity of mutation-derived neoantigens—and enabling dual T/B cell antigen discovery.^84^ MaNeo is a machine-learning platform combining random forests and extreme gradient boosting (XGBoost).^44^ Trained on immunopeptidomic data from 14 cancer types and 29 normal tissues, MaNeo constructs 406 cancer-type-specific models that integrate multi-modal features (sequence encoding, biochemical properties) to prioritize naturally presented neoantigens, reducing false positives by 30%.^44^ Jiang et al. reported deepAntigen, a GCN-based model that achieves atomic-level prediction of neoantigen-HLA binding and TCR recognition.^85^ By converting neoantigen/HLA/TCR sequences into atomic graph structures, deepAntigen captures subtle molecular interactions with an AUROC of 0.89 for neoantigen-T cell pairing, providing atomic-level guidance for vaccine optimization. The next frontier of AI-driven neoantigen research will focus on resolving remaining bottlenecks and accelerating clinical translation through several key directions.

To prevent the loss of neoantigen, several integrated strategies are emerging. Multi-region sequencing offers a more comprehensive spatial snapshot of clonal architecture and shared neoantigens, although its clinical adoption is constrained by invasiveness and practicality. Serial monitoring via circulating tumor DNA (ctDNA) enables non-invasive tracking of clonal dynamics and neoantigen persistence over time, yet current technologies often lack the sensitivity for robust neoantigen discovery, especially in low-shedding tumors or for subclonal variants. Computationally, algorithms that infer clonality from single biopsies—using variant allele frequency, tumor purity, and copy-number data—provide a more feasible alternative, although they remain inferential and may not fully capture geographically separated subclones. Moving forward, combining longitudinal liquid biopsy with deep sequencing and advanced evolutionary modeling, alongside therapeutic interventions that stabilize antigen presentation—such as epigenetic modulators or agents targeting the APM—may enhance the durability of neoantigen-directed responses and mitigate immune escape.

### Overcoming resistance and enhancing efficacy

To solve the problem of immune escape driven by neoantigen loss and immunosuppressive TME, this section focuses on strategies to remodel the TME, reverse therapeutic resistance, and amplify the antitumor efficacy of neoantigen vaccines. The efficacy of neoantigen vaccines is profoundly influenced by the immunosuppressive nature of the TME. A critical determinant is the composition and functional state of intratumoral myeloid cells. For a vaccine to succeed, APCs, particularly conventional DCs (cDCs), must efficiently capture and present neoantigens to prime T cells. However, the TME is often dominated by immunosuppressive cell types, such as myeloid-derived suppressor cells (MDSCs) and TAMs polarized toward an M2-like, pro-tumoral state. These cells can inhibit T cell priming and effector functions through mechanisms such as arginine metabolism. Furthermore, a non-inflamed TME presents a physical barrier to T cell infiltration. Therefore, overcoming this immunosuppression is likely essential for achieving robust and durable responses to neoantigen vaccination.

Targeted modulation of the immunosuppressive cells within the TME has emerged as a compelling strategy to reverse tumor immunosuppressive status and potentiate the antitumor effects of neoantigen vaccines. A promising approach is the direct *in situ* reprogramming of TAMs from a pro-tumoral M2 to an anti-tumoral M1 phenotype. This can be achieved using nanoparticles that deliver specific genetic instructions. For example, mannose-functionalized polymeric nanoparticles have been used to deliver mRNAs encoding the transcription factors IRF5 and its activator IKKβ to TAMs. This genetic reprogramming successfully shifted TAMs to an M1-like state, enhancing T cell infiltration and antitumor immunity in multiple mouse models.^86^ Moreover, injectable fibrin gel capable of the pH-responsive release of an anti-CD47 antibody was developed to block the immunosuppressive signal on cancer cells for macrophages. This localized delivery enhanced phagocytosis of tumor cells by macrophages and inhibited local recurrence and metastasis without systemic toxicity.^87^

Furthermore, strategic combination therapies are imperative to overcome this immunosuppression. The combination of neoantigen vaccines with ICIs have been proved to be necessary. Preclinical and early clinical data from mRNA vaccines such as NeoVax and BNT111 demonstrate their ability to induce robust, tumor-specific CD4^+^ and CD8^+^ T cell responses. When synergized with PD-1 blockade, this approach not only expands primed T cell clones but also reverses T cell exhaustion within the TME, markedly enhancing antitumor activity. This is evidenced by improved ORRs and PFS in malignancies such as melanoma and HNSCC. Future optimization should focus on identifying ideal combinatorial sequences, predictive biomarkers, and patient populations to maximize the clinical benefit of this synergistic approach.

In addition, radiotherapy remodels the tumor immune microenvironment by inducing ICD, releasing tumor antigens and damage-associated molecular patterns (DAMPs), and activating inflammatory signaling pathways.^63^ Accumulating evidence indicates that radiation not only activates interferon-related pathways and promotes the infiltration of CD8^+^ T cells, NK cells, and M1-polarized macrophages but also enhances antigen-presentation capacity, thereby facilitating the conversion of “cold” tumors to “hot” tumors.^88^^,^^89^ However, radiotherapy may concurrently trigger immunosuppressive mechanisms, such as upregulating PD-L1 expression and recruiting immunosuppressive cell populations.^24^ The combination of radiotherapy and neoantigen vaccines exhibits significant potential owing to their synergistic effects: (1) radiation-mediated enhancement of antigen release and presentation can amplify the T cell response primed by neoantigen vaccines^90^; (2) the radiotherapy-induced inflammatory microenvironment may augment the immunogenicity of vaccines^91^; (3) radiation can reverse immunosuppression through mechanisms such as mitophagy modulation.^92^ Future research should focus on optimizing radiation dosage and fractionation regimens to maximize immune activation,^93^ developing personalized neoantigen vaccine sequences in combination with radiotherapy, as well as exploring combinations with stromal barrier-targeted therapies for highly immunosuppressive tumors such as pancreatic cancer.

Emerging evidence underscores the critical role of specific nutrients in modulating the efficacy of tumor neoantigen vaccines. Vitamin C enhances antitumor immunity by promoting T cell infiltration into the TME and boosting the cytotoxic function of CD8^+^ T cells, showing synergistic effects with immune checkpoint blockade, particularly in mismatch repair-deficient tumors.^94^ Meanwhile, magnesium is essential for LFA-1-mediated CD8^+^ T cell co-stimulation, strengthening signal transduction, immune synapse formation, metabolic activity, and effector responses.^95^ Clinically, magnesium deficiency correlates with accelerated disease progression and reduced survival in patients receiving T cell-targeted therapies. Thus, adequate levels of these nutrients may potentiate neoantigen vaccine responses by optimizing T cell function.

### Engineering efficient neoantigen delivery systems

In direct response to the delivery-related limitations detailed in section vaccine design, this section elaborates on engineering advanced delivery systems to optimize neoantigen bioavailability, targeting specificity, and clinical translatability. The route of administration and delivery system are crucial for the vaccine’s immunogenicity. Traditional vaccines are often administered via intramuscular or subcutaneous injection, whereas novel strategies employ nanoparticles, viral vectors, or cell-membrane-coated vehicles to enable targeted delivery, thereby achieving specific enrichment of neoantigens around tumor cells. Recent advances highlight the optimization potential of microbial vectors and SANVs—two promising platforms with inherent advantages for neoantigen delivery (detailed mechanisms in sections microbial vector vaccine and nanovaccine).

**Figure 4:**
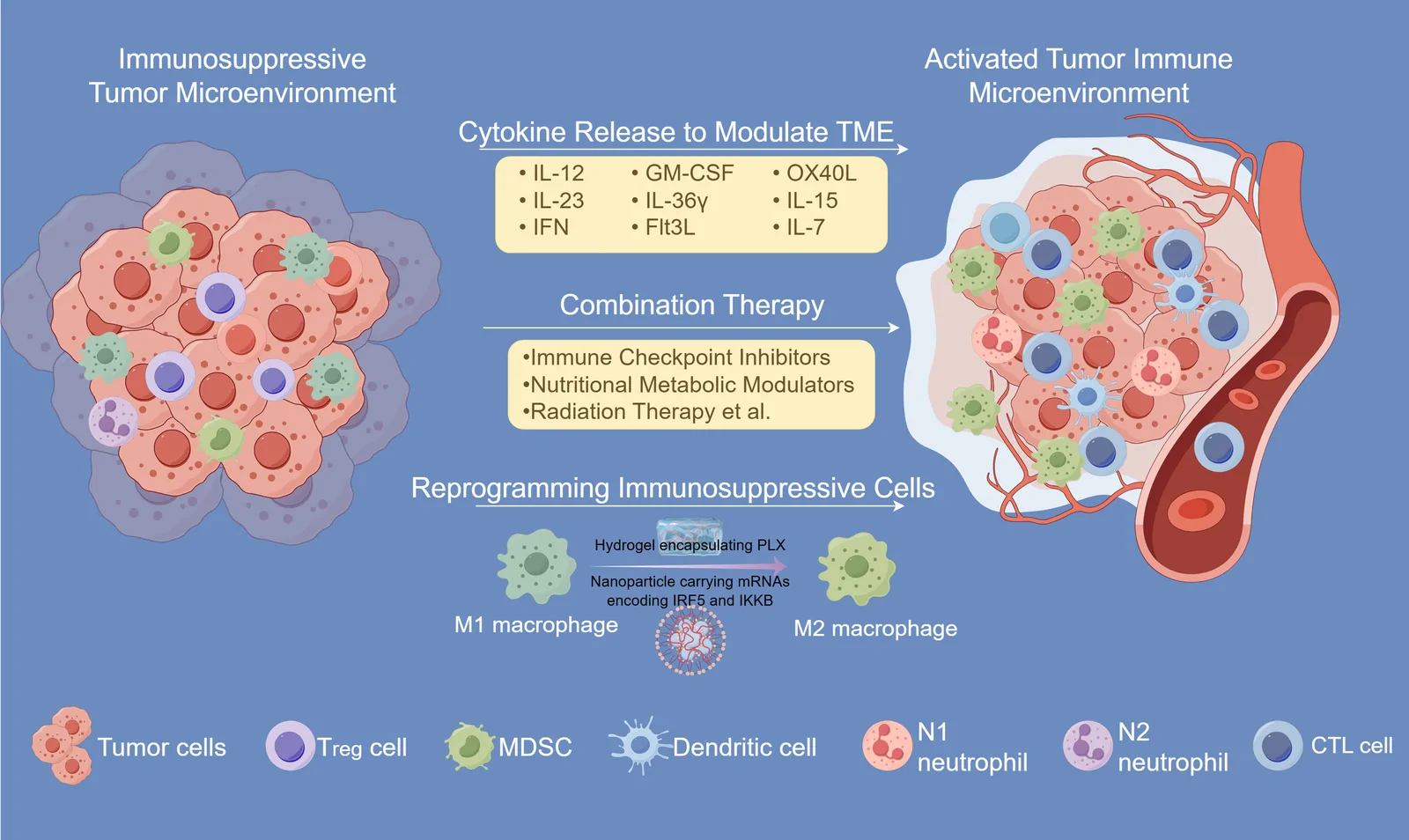
Strategies for remodeling the immunosuppressive TME to enhance neoantigen vaccine efficacy Multiple combinatorial approaches are being developed to overcome the immunosuppressive TME (TME). (1) Cytokine delivery: immunostimulatory cytokines can be delivered locally *in situ* using nanoparticles for TME modulation. This promotes DC activation and maturation, enhances antigen presentation, and fosters a pro-inflammatory TME. (2) Combination therapies: neoantigen vaccines are frequently combined with other modalities to achieve synergistic effects. These include ICIs (such as anti-PD-1 and anti-CD47), nutritional metabolic modulators, and radiation therapy, which can induce ICD and further remodel the TME. (3) Reprogramming immunosuppressive cells: the function of immunosuppressive cells, including M2-like tumor-associated macrophages (TAMs), regulatory T (Treg) cells, and myeloid-derived suppressor cells (MDSCs), can be modulated to support antitumor immunity. Strategies include using hydrogels to deliver agents such as pexidartinib (PLX) to deplete TAMs, or nanoparticles carrying mRNAs encoding transcription factors such as IRF5 and IKKβ to repolarize TAMs toward an anti-tumoral M1 phenotype. CTL, cytotoxic T lymphocyte; GM-CSF, granulocyte-macrophage colony-stimulating factor.

For nanoparticle vaccines, future delivery systems should be engineered to co-deliver neoantigens with potent adjuvants directly to APCs within the TME. A critical bottleneck is the inefficient capture and presentation of tumor-derived neoantigens by DCs. LNPs can be formulated to encapsulate mRNA encoding neoantigens alongside mRNAs for cytokines or the immunogenic parts of oncolytic viruses. Key findings are summarized in Figure 4. For instance, LNPs encapsulating the RNA genome of an oncolytic virus can successfully bypass preexisting immunity and generate viruses *in situ*, leading to potent antitumor responses.^96^ Furthermore, the activation of intracellular PRRs, such as STING, is a powerful strategy to license DCs. However, the clinical translation of STING agonists such as cyclic dinucleotides (CDNs) has been hampered by poor bioavailability and instability. Nanocarriers offer an elegant solution. As demonstrated by Dane et al., tumor-penetrating PEG-lipid nanodiscs (LNDs) can efficiently deliver CDNs deep into tumor tissues, protecting them from degradation and potently priming anticancer immunity.^97^

For microbial vectors, key optimization directions focus on addressing core limitations while retaining their intrinsic tumor tropism and immunostimulatory properties. Two primary utilizable types have emerged: whole bacterial cells and bacteria-derived nanovesicles. As described in section nanovaccine, engineered probiotic bacteria serve as efficient live vectors that can be programmed to produce and deliver tumor-specific neoantigens directly to the immune system. This approach leverages the natural tropism of bacteria for TMEs and their inherent immunostimulatory properties to induce potent, specific T cell responses. Alternatively, outer-membrane vesicles (OMVs)—nanoparticles naturally secreted by bacteria—represent a promising acellular strategy. Packed with PAMPs, OMVs act as potent adjuvants to induce trained immunity. They prime innate immune cells by activating inflammasome pathways, thereby creating a heightened state of alert that dramatically enhances the efficacy of subsequent tumor antigen vaccination. Together, these strategies utilize the unique immunostimulatory features of microbes to develop more effective and precise cancer immunotherapies.^26^

## Future perspective

Neoantigen vaccines, a crucial breakthrough in precision cancer immunotherapy, exhibit significant clinical value and broad future prospects. These vaccines show differential but complementary value across disease stages. In early-stage cancers, vaccines targeting clonal neoantigens—shared across all tumor cells—may prevent recurrence by eliminating minimal residual disease, leveraging the relatively intact immune system. For advanced/metastatic cases, where tumors display high heterogeneity, vaccines often combine clonal and subclonal neoantigens. This addresses diverse subpopulations, overcoming immune evasion. Tailoring neoantigen selection to disease progression enhances efficacy, making these vaccines a promising strategy across the cancer continuum.

Neoantigen vaccine development is inherently a convergence science endeavor, and this interdisciplinary integration serves as the core logical backbone for future research breakthroughs. Computational biology empowers high-throughput identification and prioritization of tumor-specific epitopes from complex multi-omics data expanding the targetable neoantigen repertoire beyond conventional mutation-based approaches. Immunology provides the foundational framework for deciphering antigen-presentation T cell activation and immune memory guiding the selection of epitopes with high immunogenic potential and enabling rational vaccine design for robust durable antitumor responses. Materials science, particularly advances in nucleic acid delivery systems such as SANVs and lipid nanoparticles ensures stable encapsulation, efficient *in vivo* delivery, and controlled release of neoantigen payloads, directly enhancing immunogenicity and safety. Clinical trial design bridges these foundational disciplines by translating preclinical insights into rigorously designed studies optimizing patient stratification based on multi-omics profiles refining combination-therapy regimens and validating interdisciplinary innovations in real-world clinical settings. Together, these four fields form a synergistic ecosystem that transforms neoantigen discovery from computational prediction to clinically viable immunotherapy. For instance, in the study by Ji et al., integration of FL-RNC-seq-based epitope discovery (rooted in computational biology) and AI-driven immunogenicity prediction (as an immunology-informed computational tool) with LNP-formulated mRNA vaccines (an advance in materials science) and preclinical validation of synergy with PD-1 blockade (as a precursor to clinical trial design) achieved potent tumor control. This vividly demonstrates how cross-disciplinary collaboration overcomes single-discipline limitations.^28^ Framing neoantigen vaccine development through the lens of convergence science will be essential to systematically address existing bottlenecks from inaccurate neoantigen prediction to immunosuppressive TME and fully unlock the potential of personalized cancer immunotherapy, ultimately enabling broader clinical application across diverse tumor types.

Recent advances in HTS technologies and sophisticated bioinformatics tools have significantly promoted the rapid identification of neoantigens and the development of PCVs. These cutting-edge approaches enable efficient screening of tumor-specific mutations, accurate prediction of immunogenic peptides, and optimization of vaccine design. By integrating multi-omics data and AI-driven predictive models, researchers can better tailor therapeutic strategies to individual patients, enhancing antitumor immune responses. These breakthroughs not only accelerate the clinical translation of neoantigen-based immunotherapy but also pave the way for more precise and effective cancer treatment paradigms in the future.^28^

In order to enhance immunogenicity and treatment outcomes in cancer immunotherapy, remarkable progress has been witnessed in the innovation of vaccine types and delivery systems. Novel mRNA vaccines, inspired by the success of COVID-19 mRNA vaccines, have shown great promise. Their ability to precisely encode tumor neoantigens enables the induction of robust immune responses, as demonstrated by their significant impact in certain cancer trials. Additionally, new microbial vectors, such as engineered probiotics, are being explored. These vectors can be optimized to efficiently deliver neoantigens and trigger antitumor immunity.^68^ However, challenges persist, including accurate neoantigen prediction and ensuring widespread applicability. Despite these hurdles, the advancements in these two aspects open up new avenues for more effective and personalized cancer immunotherapy, warranting continued research and development efforts.

## Funding and acknowledgments

This work was supported by the 10.13039/501100001809National Natural Science Foundation of China (82403874), the 10.13039/501100002858China Postdoctoral Science Foundation (2023M741629 and 2024T170397), the Jiangsu Funding Program for Excellent Postdoctoral Talent (2024ZB062), the 10.13039/501100004608Natural Science Foundation of Jiangsu Province (BK20241203), and the Medical Key Science and Technology Development Project (ZKX23017) of the Nanjing Municipal Health Commission, Study on the Management Model of Chronic Disease Prevention and Control in Medical Institutions (YM2024ZD028). All illustrations in Figures 1, 2, 3, and 4 were generated by Figdraw.

## Author contributions

X.W. and L.W. investigated and summarized the literature. X.W., L.W., and F.C. formulated the concept and structure of the paper. X.W., S.S., and S.T. generated the figure sets. X.W., L.W., S.W., X.C., and F.C. wrote and edited the manuscript. L.W. and F.C. supervised the work and applied for funds. All authors contributed to the manuscript and approved the final version.

## Declaration of interests

The authors declare no competing interests.
